# Direct Synthesis of Molybdenum Phosphide Nanorods on Silicon Using Graphene at the Heterointerface for Efficient Photoelectrochemical Water Reduction

**DOI:** 10.1007/s40820-021-00605-7

**Published:** 2021-03-01

**Authors:** Sang Eon Jun, Seokhoon Choi, Shinyoung Choi, Tae Hyung Lee, Changyeon Kim, Jin Wook Yang, Woon-Oh Choe, In-Hyuk Im, Cheol-Joo Kim, Ho Won Jang

**Affiliations:** 1 Department of Materials Science and Engineering, Research Institute of Advanced Materials, Seoul National University, Seoul, 08826 Republic of Korea; 2 Department of Chemical Engineering, Pohang University of Science and Technology, Pohang, 37673 Republic of Korea

**Keywords:** Photoelectrochemical water splitting, Silicon, Molybdenum phosphide, Hydrogen evolution, Graphene

## Abstract

**Highlights:**

- MoP nanorod-array catalysts were directly synthesized on graphene passivated silicon photocathodes without secondary phase.
- Mo-O-C covalent bondings and energy band bending at heterointerfaces facilitate the electron transfer to the reaction sites.
- Numerous catalytic sites and drastically enhanced anti-reflectance of MoP nanorods contribute to the high solar energy conversion efficiency.

**Abstract:**

Transition metal phosphides (TMPs) and transition metal dichalcogenides (TMDs) have been widely investigated as photoelectrochemical (PEC) catalysts for hydrogen evolution reaction (HER). Using high-temperature processes to get crystallized compounds with large-area uniformity, it is still challenging to directly synthesize these catalysts on silicon photocathodes due to chemical incompatibility at the heterointerface. Here, a graphene interlayer is applied between p-Si and MoP nanorods to enable fully engineered interfaces without forming a metallic secondary compound that absorbs a parasitic light and provides an inefficient electron path for hydrogen evolution. Furthermore, the graphene facilitates the photogenerated electrons to rapidly transfer by creating Mo-O-C covalent bondings and energetically favorable band bending. With a bridging role of graphene, numerous active sites and anti-reflectance of MoP nanorods lead to significantly improved PEC-HER performance with a high photocurrent density of 21.8 mA cm^−2^ at 0 V versus RHE and high stability. Besides, low dependence on pH and temperature is observed with MoP nanorods incorporated photocathodes, which is desirable for practical use as a part of PEC cells. These results indicate that the direct synthesis of TMPs and TMDs enabled by graphene interlayer is a new promising way to fabricate Si-based photocathodes with high-quality interfaces and superior HER performance.

**Graphic Abstract:**

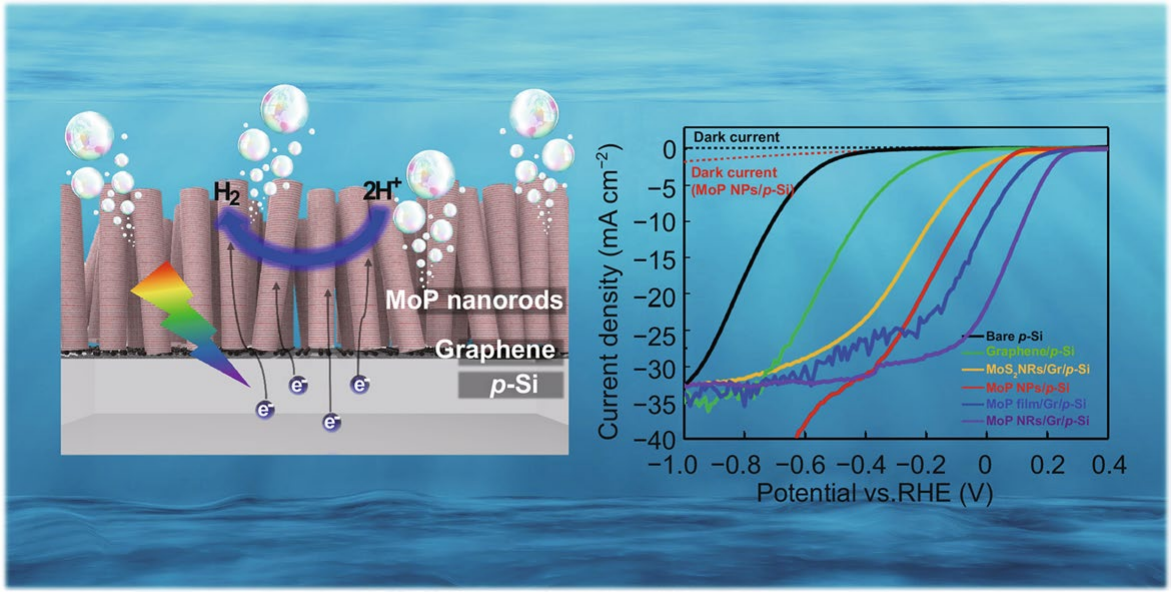

**Supplementary Information:**

The online version contains supplementary material available at (10.1007/s40820-021-00605-7).

## Introduction

Hydrogen is expected to become sustainable future energy owing to zero-emission, non-toxicity, and large energy density [1]. However, it usually exists in covalent compounds combined with other elements such as oxygen, nitrogen, and organic materials. One of the promising methods to obtain pure hydrogen from these compounds is the coupling of solar energy to electrochemical water splitting, such as photovoltaic-electrochemical (PV-EC), and photoelectrochemical (PEC) water splitting [2, 3]. Out of two systems, a single cost-effective device can be achieved by PEC water splitting which efficiently converts intermittent and unpredictable solar energy to chemical binding energy [4]. Silicon and III–V semiconductors have been extensively studied for photoabsorbers in PEC water splitting system [5–7]. Among them, p-type Si is one of the most promising photocathodes due to its earth abundance, advanced infrastructure, high carrier mobility, and suitable band gap, which closely matches the solar spectrum [8, 9]. However, it has the problem of insufficient output photovoltage arisen from its narrow band gap limited by the quasi-Fermi level difference at the interfaces under full illumination [10]. Also, the surface of p-type Si is catalytically inactive due to its high hydrogen adsorption Gibbs free energy (∆*G*_H_). The other problem is that it suffers from photo-corrosion in aqueous solution because of the thermodynamical reduction potential of silicon adjacent to water reduction potential (H+/H_2_) [11].

By employing photoelectrochemical catalysts, both the interfacial reactions can be expedited via lowering the activation energy, and the silicon can be protected from photoactive dissolution [12]. Recently, transition metal phosphides (TMPs) and transition metal dichalcogenides (TMDs) have received tremendous attention because of strong interaction with solar irradiation, tunable optoelectronic properties, and abundant reaction sites for hydrogen evolution reaction (HER) [13–17]. In our previous studies, thickness-optimized 2D MoS_2_ [18], WS_2_ [19], and sulfur-doped MoP thin-film catalysts [20] synthesized by a simple in-vacuum thermolysis process exhibited remarkable catalytic activity and stability as PEC catalysts. Also, various transition metal-based catalysts synthesized by a vacuum process, such as NbS_2_ [21], MoS_2_/WS_2_/WSe_2_ [22], MoQ_x_Cl_y_ (*Q* = *S*, Se) [23], and CoP [24] have been widely investigated. However, with the high-temperature sulfurization or phosphorization process, which is well-known synthetic methods for highly uniform, scalable, and crystalline transition metal-based catalysts, there is a big issue in the direct synthesis of the catalysts just above the silicon photocathodes because they are chemically incompatible, leading to the formation of secondary compounds such as metal silicides. The inevitably formed secondary phase acts as a parasitic light absorption layer that causes the loss of photon reaching the silicon photocathode. Also, its low shunt resistance reduces catalytic activity by allowing light-generated electrons to move in an alternative path rather than to the surface of the catalyst. For example, Benck et al. [25] directly deposited Mo layers on p-Si using DC magnetron sputter, followed by sulfurization. However, inevitably formed interlayers such as Mo and Mo_x_Si induced the parasitic light absorption resulting in the low photocurrent density. Also, Hasani et al. [26] developed a hybrid sulfurization method of depositing MoO_3_ film on p-Si, spin-coating (NH_4_)_2_MoS_2_ on it, and converting them into MoS_2_ film. Even though the uniformly distributed and thickness-controlled thin films are synthesized, the photocathodes exhibited leakage current density arising from interfacial secondary phase between silicon and MoS_2_ [27, 28]. As a result, it is essential to apply a novel method that prevents the formation of metallic secondary phase acting as parasitic light absorption layer and providing inefficient electron path for hydrogen evolution reaction.

Here, we demonstrate the direct growth of highly uniform MoP catalysts on p-Si photocathodes by applying the graphene interlayer at the heterointerface. As graphene completely suppresses the chemical reaction between the silicon and Mo atoms, the Mo_x_Si layer does not exist. As a result, most of the incident light can be delivered to silicon photocathodes as graphene only absorbs 2.8–4.3% of visible light [29]. Also, no alternative current path between the silicon and catalyst is provided, leading to the absence of leakage current and enhanced catalytic activity. Furthermore, Mo-O-C covalent bondings, which connects graphene and MoP, is favorably formed to accelerate the electron transfer at heterointerface. Moreover, the large work function difference at the graphene/p-Si interface contributes to a strong band bending, facilitating the electron transfer. Based on these advantages of the graphene acting as a bridge between p-Si and MoP, vertically aligned MoP nanorods (NRs) are directly synthesized on silicon photocathode. To make good use of numerous catalytic sites ascribed to P atoms, we designed a one-dimensional nanostructured MoP with a high surface-to-volume ratio [30, 31]. In addition, drastically enhanced anti-reflectance of nanorod-arrays contributes to the overall light absorption of photocathodes. With an optimized nanorod-arrays thickness of 100 nm, the photocathode exhibits a remarkable photocurrent density of 21.8 mA cm^−2^ at 0 V versus a reversible hydrogen electrode (RHE) and enhanced long-term stability. While MoS_2_ NRs/graphene/p-Si shows a dramatic degradation with increasing pH, MoP NRs/graphene/p-Si was considerably stable to changes in pH. Besides, it maintained high PEC performance with increasing electrolyte temperature up to 70 ℃. Using graphene interlayer, not only MoP but also various catalysts such as nickel phosphide and cobalt phosphide can be directly synthesized on silicon photoelectrode. This study demonstrates the huge potential of the graphene to solve the interfacial incompatibility and will contribute to a large-scale direct synthesis of nanostructured transition metal-based materials exhibiting an efficient hydrogen production performance.

## Experimental

### Synthesis of Graphene

Monolayer graphene film was grown by chemical vapor deposition on Cu foil (Nilaco corporation, #CU-113213, 30 μm thick, 99.9% purity). First, the Cu foil was annealed at 1030 °C for 4 h under flow of H_2_ at 70 sccm with a total pressure of 5 Torr; then, monolayer graphene film was grown at 1040 °C for 2 h by additionally introducing 1% CH_4_ diluted in H_2_ at 2 sccm. During the cooling process after growth, the 1% CH_4_ diluted in H_2_ gas injection was kept until the film had cooled to 600 °C, then only H_2_ was introduced until the temperature reached under 150 °C to allow unloading of the sample.

### Fabrication of MoP NRs/Graphene/p-Si Photocathodes

The boron-doped (100) p-type silicon wafers (1–10 Ω cm) for MoP nanorods(NRs)/graphene(Gr)/p-Si photocathodes and the conductive arsenic-doped (100) n-type silicon wafers (0.001–0.005 Ω cm) for MoP NRs/Gr/n^++^-Si were cut into pieces with a size of 1.5 × 1.5 cm^2^. The silicon pieces were cleaned using acetone, isopropyl alcohol, and DI water by ultrasonication for 15 min at each step. To remove the native oxide layer, cleaned Si wafers were soaked into HF for 30 s. The PMMA/graphene layer was transferred onto the silicon surface and PMMA was removed by acetone at 60 ℃ for 15 min. MoP NRs/Gr/p-Si photocathodes were fabricated by MoO_3_ precursor (Taewon Co.) deposition and high-temperature phosphorization process. Vertically aligned molybdenum trioxide nanorods with 100 nm thickness were deposited on graphene/p-Si photocathodes by glancing angle deposition (GLAD) electron beam evaporator. The substrates were tilted at an angle of 80° and rotated with a speed of 80 rpm. The base pressure and growth rates were 1.0 × 10^–6^ mTorr and 1.5 Å s^−1^, respectively. By simple phosphorization process, MoO_3_ nanorods turned into MoP nanorods. High-purity H_2_ gases mixed with N_2_ gases were utilized for this process in a thermal chemical vapor deposition (CVD) system. At first, the temperature of the CVD chamber was increased to 500 ℃ and maintained for 15 min under the flow of H_2_ and N_2_ at 0.85 Torr. The flow rate of H_2_ and N_2_ was 100 and 500 cm^3^ min^−1^, respectively, measured by mass flow controllers. The temperature was increased to 900 ℃ maintaining for 15 min. The sublimation of phosphorus powder (Sigma-Aldrich, 99.9% purity) proceeded in the other heating zone. The temperature of the sublimation region was set at 400 ℃ and 0.7 g of powder was used.

### Fabrication of MoP film/Graphene/p-Si and MoP NPs/p-Si Photocathodes

The MoO_3_ film with intended thickness of 5 nm was deposited on graphene/p-Si and HF-treated bare p-Si for the fabrication of MoP film/Gr/p-Si and MoP NPs/p-Si, respectively. During deposition, the substrates were kept horizontal without rotation. The base pressure and growth rates were 1.0 × 10^–6^ mTorr and 0.1 Å s^−1^, respectively. After film deposition, the phosphorization process with the temperature of 700 ℃ in H_2_ and N_2_ (100 and 500 cm^3^ min^−1^) atmosphere proceeded for 15 min. The other heating zone where 0.7 g of phosphorus powder sublimates was maintained at 400 ℃.

### Fabrication of Various TMPs and TMDs/Graphene/p-Si Photocathodes

The metal oxide films of CuO, WO_3_, Fe_2_O_3_, CoO_3_, and NiO with intended thickness of 5 nm were deposited on graphene/p-Si for the synthesis of CuP_x_, WP_x_, FeP_x_, CoP_x_, and NiP_x_ catalysts. Also, MoO_3_ and WO_3_ film with 5 nm thickness were deposited to synthesize MoS_2_ and WS_2_ on graphene/p-Si. During the phosphorization and sulfurization process, the temperature of the CVD furnace was maintained at 700 ℃ for CuP_x_, CoP_x_, WS_2_, and 900 ℃ for WP_x_, FeP_x_, NiP_x_, MoS_2_ catalysts. The temperature of the sublimation region was set at 400 ℃ with 0.7 g of phosphorus powder and 300 ℃ with 0.7 g of sulfur powder.

### Materials Characterization

The Raman spectra were obtained using Lab RAM HR (Horiba JobinYvon, Japan) with 532 nm excitation wavelength. The FT-IR spectra measurements were conducted with Nicolet iS50 (Thermo Fisher Scientific). The surface morphology and cross-sectional images were acquired by FE-SEM (MERLIN COMPACT, JEISS). Low magnification, high-resolution images, and EDS data were obtained with TEM (JEM-2100F, JEOL). The chemical bonding states and work function of MoP nanorods/graphene/p-Si were characterized by XPS (AXIS SUPRA, Kratos). The crystal structures of MoP film and MoP nanorods were analyzed by XRD (D8 discover, Bruker). The reflectance and absorption spectra were obtained with UV–visible spectroscopy (V-770, JASCO). The electrical properties of the deposited catalysts were measured by an Agilent 4156C semiconductor analyzer.

### Photoelectrochemical Measurements

The photoelectrochemical properties of as-synthesized photocathodes were measured with a potentiostat (Ivium Technologies, Nstat) using a three-electrode system consisting of a saturated calomel electrode (SCE) as the reference electrode and a graphite rod as the counter electrode. 0.5 M H_2_SO_4_ standard solution was used as an electrolyte. A quartz vessel showing high transmittance for UV wavelengths of 200 nm or less was utilized. A Xe arc lamp calibrated to 100 mW cm^−2^ (AM 1.5 G condition) was used as a light source. In the process of the linear sweep voltammogram (LSV) measurements, the photocathodes were polarized at a scan rate of 10 mV s^−1^ under AM 1.5 G. The IPCE data were obtained with a monochromator (MonoRa150) and the potential of 0 V vs. RHE was applied. Electrochemical impedance spectroscopy (EIS) was carried out with the frequency from 250 kHz to 1 Hz using 10 mV alternating current.

## Results and Discussion

### Graphene Interlayer for Enhanced Light Absorption and Catalytic Activity

**Fig. 1:**
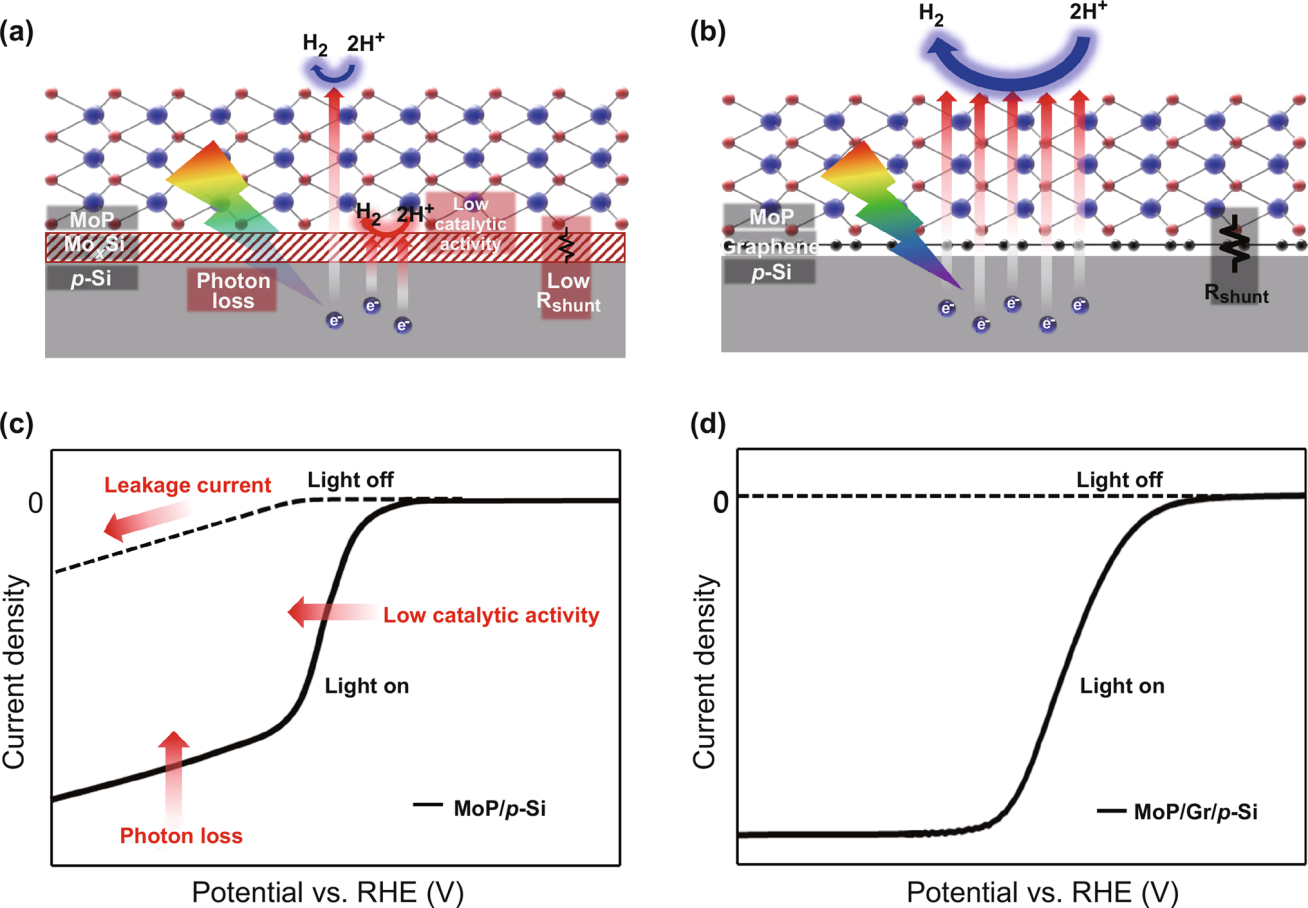
Schematic illustrations of MoP synthesized on **a** bare p-Si with Mo_x_Si and **b** graphene passivated p-Si. Linear sweep voltammograms of **c** MoP/p-Si photocathode with photon loss and low catalytic activity and **d** MoP/graphene/p-Si photocathode showing enhanced PEC performance

In Fig. 1a, b, schematic illustrations of photocathodes with MoP catalysts synthesized on bare p-Si and graphene passivated p-Si are shown, respectively. As shown in Fig. 1a, the Mo_x_Si layer, a secondary compound of silicon and Mo atoms, is laterally formed between p-Si and MoP catalyst. It induces parasitic light absorption resulting in a reduction of photogenerated charges. In addition, due to its low shunt resistance, some of the light-generated electrons are trapped in the metallic layer and can not reach the MoP catalyst. As unintended hydrogen evolution reaction occurs at the secondary phase with no suitable reaction sites, the photocathode shows low catalytic activity and PEC performance. One possibility for hydrogen production to be active in silicide layer may be a spillover effect in which hydrogen species adsorbed to reaction sites with negative Δ*G*_H_ diffuse to the sites with positive Δ*G*_H_ [32]. However, Δ*G*_H_ of *P* sites, the most catalytic sites of MoP, changes from negative to positive when H coverage increases, which means that as soon as the hydrogen is adsorbed, it is desorbed immediately before the spillover effect occurs [33]. As a result, active proton reduction from the spillover effect can not occur in silicide layer. On the other hand, as shown in Fig. 1b, both the reduced photon loss and enhanced HER activity can be achieved by graphene interlayer at a heterointerface. Linear sweep voltammograms clearly identify the contributrion of graphene to PEC performance of heterostructure photocathodes in Fig. 1c, d. With the direct synthesis of MoP on bare p-Si, the reduction of saturation current density can be observed in Fig. 1c, consistent with photon loss derived from the metallic layer. Also, a certain amount of leakage current with no light illumination clarifies the existence of a conducting layer having low shunt resistance. Because of decreased electrons involved in proton reduction at MoP catalyst, the onset potential of the LSV curve shows a shift toward a cathodic direction. On the contrary, large saturation current density and lowered onset potential with no leakage current can be achieved by the photocathode to which the graphene interlayer is applied in Fig. 1d.

### Characterization of MoP Nanorods/Graphene/p-Si Photocathode

**Fig. 2:**
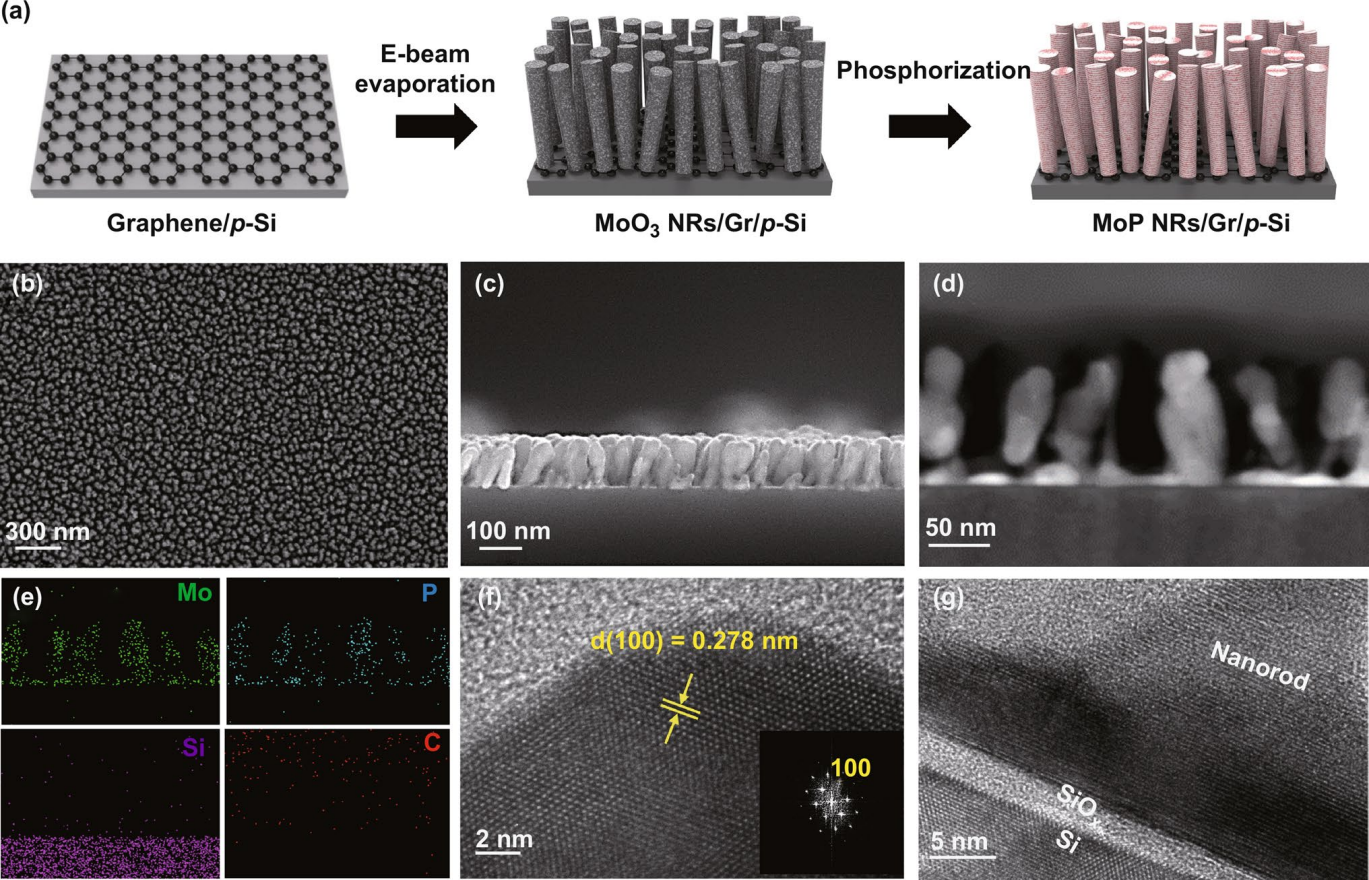
**a** Schematic of the synthesis method. **b** SEM image (top-view) of MoP nanorods. **c** Cross-sectional SEM image of MoP nanorods. **d** Cross-sectional STEM image and **e** EDS element maps of Mo, P, Si, and C for MoP nanorod-arrays. **f** High magnification TEM images of MoP nanorod with (100) plane and corresponding fast Fourier transformation (FFT) pattern. **g** Cross-sectional TEM image of MoP NRs/graphene/SiO_x_/p-Si surface region

Figure 2a shows a schematic illustration of MoP nanorods(NRs)/graphene(Gr)/p-Si photoelectrode fabrication (see the Experimental Section and Fig. S1 for details). Graphene having tightly packed carbon atoms with an sp^2^-hybridized lattice is grown on copper foil with large grain size (Fig. S2a) [34]. It is transferred to the p-Si substrate and completely covers the surface of silicon (Fig. S2b). Using electron beam evaporator, MoO_3_ nanorods are deposited on graphene with a thickness of 100 nm. MoP nanorod-array catalysts are finally synthesized on graphene passivated silicon wafer by vapor phase phosphorization of MoO_3_ precursor. As for the comparative group, MoP nanoparticles and MoP film are synthesized on p-Si and graphene/p-Si, respectively (see the Experimental Section for details). The photographic images of centimeter-scale Graphene/p-Si and MoP NRs/Gr/p-Si is represented in Fig. S3a, b. It indicates that the graphene monolayer is uniformly transferred to p-Si substrates, and MoP nanorod-arrays are successfully grown on graphene.

The vertically aligned nanorod structure is identified by SEM images. In Fig. 2b, the top-view SEM image of MoP nanorods is shown. It is confirmed that each MoP nanorod has a strictly uniform diameter (~ 20 nm) and is well-separated as glancing angle deposition (GLAD) induces a self-shadowing effect leading to homogeneous MoP nucleation [35]. The morphologies of MoP nanoparticles(NPs)/p-Si and MoP film/Gr/p-Si are shown in Fig. S4. As we can see in Fig. 2c, the thickness of the MoP NRs layer is 100 nm, which is quite thick compared to the thin-film catalysts. Even though a certain amount of incident light can be interrupted, it is compensated by the anti-reflectance of nanorod-arrays leading to no significant decrease in the overall amount of light absorption [36]. It will be identified by the reflectance vs. wavelength spectra. Meanwhile, what we aimed to achieve from nanorod structure is a high surface-to-volume ratio. In other words, by increasing the catalytically active area with a structural approach [37], the photoelectrochemical performance of MoP NRs/Gr/p-Si photocathode is enhanced compared to that of MoP film/Gr/p-Si photocathode. As MoP has numerous active sites ascribed to P atoms exhibiting low ΔG_H_ and acting as “H delivery” [33, 38], increasing the surface area of MoP is a key strategy to obtain a high photocurrent density. The constituent elements and atomic structure of the synthesized MoP are investigated by transmission electron microscopy (TEM). In Fig. 2d, e, uniformly distributed Mo and P elements with Si are shown by scanning TEM (STEM) image and the EDS elemental mapping images. As shown in Fig. 2f, a high-resolution TEM image (HR-TEM) and fast Fourier transformation (FFT) pattern indicate the presence of highly-crystallized MoP nanorod. The clear and well-defined lattice fringes with a d-spacing of 0.278 nm and corresponding fast Fourier transform are consistent with the (100) plane for hexagonal MoP [39]. The high-resolution cross-sectional TEM image in Fig. 2g represents that MoP nanorod with high-crystallinity is formed just above the graphene-passivated silicon substrate. In Fig. S5, the existence of graphene interlayer can be identified by zoom-in cross-sectional HR-TEM image. All of the MoO_3_ precursors are successfully transformed into MoP catalysts without remaining MoO_3_ layers. Moreover, the formation of Mo_x_Si layers is prevented by using graphene passivation.

The surface constituents and chemical nature of MoP nanorods and film are further investigated by X-ray photoelectron spectroscopy (XPS). The full XPS spectra show the existence of Mo, P, O, and C (Fig. S6a). The XPS profiles of Mo 3d and P 2p of both MoP film and MoP nanorods are presented in Fig. S6b and c. Two pairs of doublets at 231.5 eV/228.4 eV (Mo^3+^ 3d_3/2_/Mo^3+^ 3d_5/2_) and 130.5 eV/129.6 eV (P 2p_1/2_/P 2p_3/2_) are ascribed to MoP [38], while the rest of the peaks corresponds to MoO_3_, MoO_2_, and PO_4_^3−^ caused by surface oxidation [33].

**Fig. 3:**
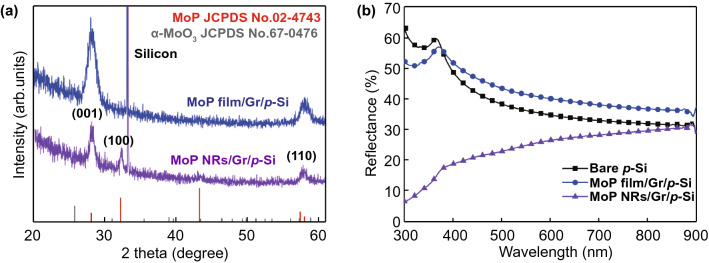
**a** XRD spectra. **b** Reflectance versus wavelength spectra of bare p-Si, MoP film/Gr/p-Si, and MoP NRs/Gr/p-Si

In Fig. 3a, X-ray diffraction (XRD) patterns of crystalline MoP film and nanorod-arrays on graphene/p-Si are provided. Characteristic peaks of MoP film are observed at 28° and 58°, which correspond to the (001) and (110) facets of MoP, respectively [33]. In the case of MoP nanorods, an additional peak at 32° corresponding to the (100) facets is identified, matching the result of the HR-TEM image. In both samples, the peak corresponding to MoO_3_ phase is not observed. The optical reflectance and absorbance of bare p-Si, MoP film/Gr/p-Si, and MoP NRs/Gr/p-Si photocathodes in the wavelength range from 300 to 900 nm are shown in Figs. 3b and S7a. MoP film grown on graphene/p-Si exhibits about 5% higher reflectance than the bare silicon in all wavelengths. However, even the same materials, the reflectance values of nanorod-structured MoP are much lower than that of film structure. It has a reflectance of 30% at a wavelength of 900 nm, and the value becomes smaller as the wavelength decreases, showing about 6% reflectance at a wavelength of 300 nm. Due to the porous nature of the material, MoP nanorod-arrays have a lower refractive index weakening broadband light reflectance [40, 41]. Such an outperforming anti-reflectance of MoP NRs/Gr/p-Si photocathode can contribute to the enhanced solar energy conversion efficiency. For MoP NPs/p-Si photocathode in Fig. S7b, though there is photon loss induced by metallic Mo_x_Si layer, it seems that MoP nanoparticles significantly affect light absorption and there is no big difference in the reflectance compared to MoP film/Gr/p-Si.

**Fig. 4:**
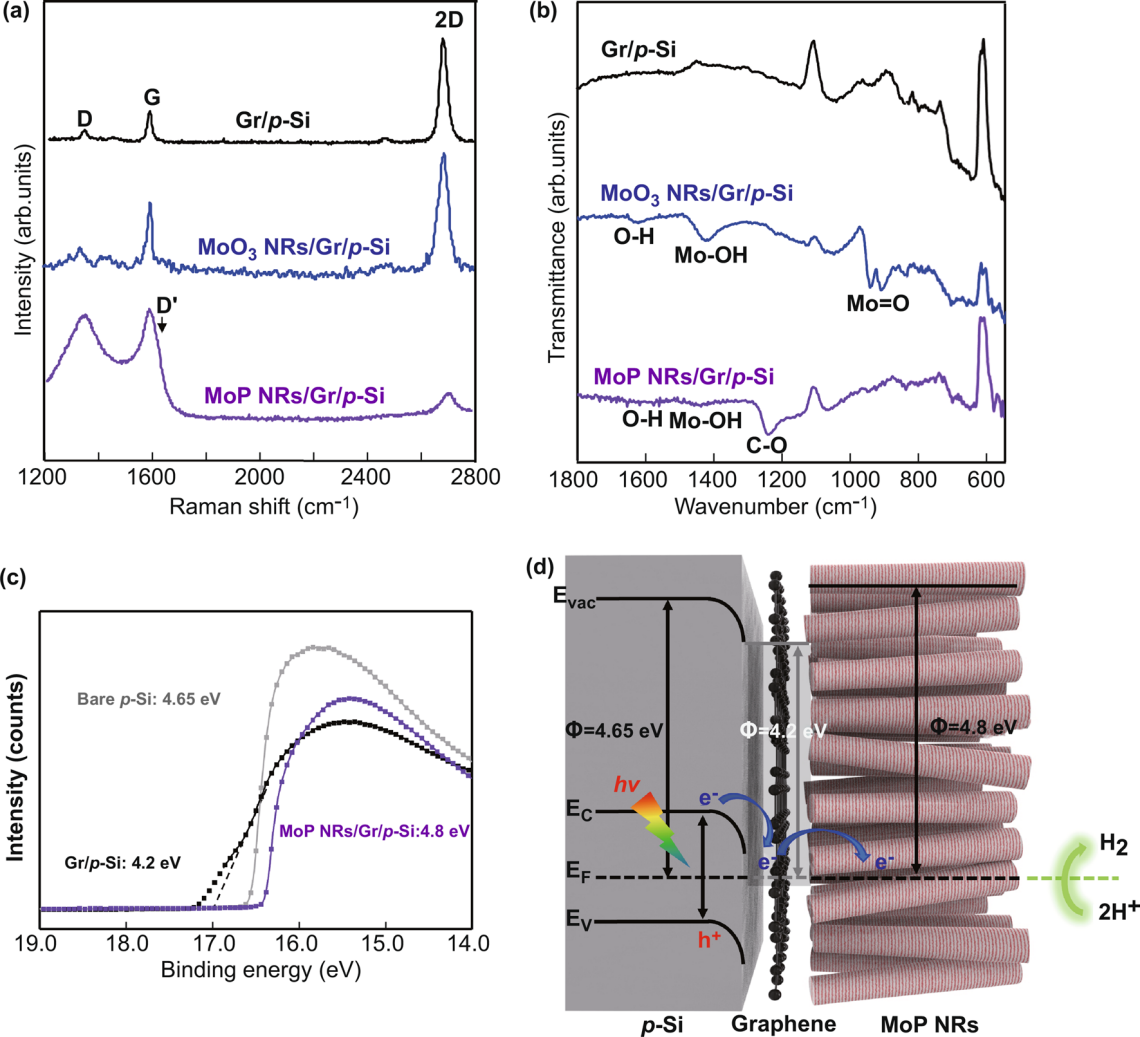
**a** Raman spectra of graphene when transferred to a p-Si wafer, covered with MoO_3_ nanorods precursor, and covered with MoP nanorods catalysts. **b** FT-IR spectra. **c** Ultraviolet photoemission spectroscopy (UPS) as a function of binding energy for the work function of p-Si, graphene/p-Si and MoP NRs/graphene/p-Si. **d** Schematic of the energy band diagram showing favorably formed band bending between p-Si and graphene

As the graphene is a pathway through which photogenerated electrons must pass until they reach the electrolyte, it is necessary to identify how the graphene chemically and electrically interacts with MoP and silicon. At first, to characterize the chemical interaction between the graphene and MoP, Raman and FT-IR spectra were investigated in each of the three steps where the MoP NRs/Gr/p-Si photocathode is fabricated. In Fig. 4a, Raman spectra show three distinct peaks of D peak at 1350 cm^−1^, G peak at 1590 cm^−1^, and 2D peak at 2680 cm^−1^, which originated from the out of plane vibrations, the in-plane vibrations of *sp*^2^-bonded carbon atoms, and the second-order two phonon process, respectively [42]. As for Gr/p-Si and MoO_3_ NRs/Gr/p-Si samples, typical *I*_G_/*I*_2D_ peak ratio of monolayer graphene and the negligible D peaks are observed, implying a well-progressed graphene transfer and MoO_3_ precursor deposition. However, after phosphorization, the intensity of D peak is drastically increased while that of 2D peak is decreased. It is well known that defects are required to activate D peak and deactivate 2D peak of graphene [43]. Specifically, *sp*^3^-type defects are likely introduced since D' peak is not visible [44]. Among some kinds of *sp*^3^-type defects, it is most likely that oxygen-containing functional groups are formed by receiving oxygen from the MoO_3_ precursor as thermal energy was applied to the interface between graphene and MoO_3_ during the heating process. To clearly verify that this Raman spectrum is derived from the bonding with oxygen, not affected by H_2_ annealing or gas phase phosphorus, Raman spectra of Gr/p-Si and MoO_3_ NRs/Gr/p-Si photocathodes after annealing at 900 ℃ in H_2_/N_2_ atmosphere are shown in Fig. S8a and b, respectively. For Gr/p-Si photocathode after annealing, *I*_G_/*I*_2D_ peak ratio increased, which attributes to the generation of defects caused by hydrogen etching [45]. However, *I*_D_/*I*_2D_ peak ratio is still small which is not appropriate to explain the Raman spectra of MoP NRs/Gr/p-Si. In Fig. S8b, although there is no flowing of phosphorus during the annealing, the same Raman spectrum with MoP NRs/Gr/p-Si was obtained. From this result, it is confirmed that interfacial bonding between graphene and MoO_3_ are formed due to high thermal energy.

To further investigate the presence of the functional group in graphene, the samples are analyzed by FT-IR spectroscopy in Fig. 4b. Unlike graphene oxide and reduced graphene oxide, monolayer graphene shows no C=C aromatic stretching [46]. As for MoO_3_ NRs/Gr/p-Si sample, the bands at 943 cm^−1^ and 910 cm^−1^ are apparent, attributed to Mo=O characteristic vibrations of the orthorhombic phase MoO_3_ [47, 48]. After phosphorization, with the MoP NRs/Gr/p-Si sample, MoO_3_ precursor has been completely converted to MoP as can be seen from the disappearance of Mo=O peaks and the most discernible band appears at 1242 cm^−1^ corresponding to C–O epoxy stretching [49–51]. The peaks at 1422 and 1625 cm^−1^ are related to the vibration of the Mo-OH bond and the bending of adsorbed water, respectively [52, 53]. Judging from the existence of C–O vibration, it appears that oxygen is bound to graphene interlayer leading to the formation of Mo-O-C covalent bondings at the interface between graphene and MoP NRs. Considering that flowing hydrogen gas for phosphorization induces graphene etching and the broken C–C bonds appear with destroyed carbon lattice [45], it is likely that the broken bonds in lattice function as reactive sites and contribute to covalent bondings such as Mo-O-C under high-temperature condition [54]. Consequently, as strong chemical interaction can be achieved by covalent bondings, the photogenerated electrons transport easily at the graphene/MoP NRs interface with low interfacial charge resistance, enhancing the photoelectrochemical performance.

Secondly, the electrical interaction between graphene and silicon photocathode is analyzed using ultraviolet photoelectron spectroscopy (UPS) to demonstrate the band alignment of heterostructure in hydrogen production. In Fig. 4c, the secondary electron emission (SEE) cutoffs as a function of binding energy are shown for the work function of p-Si (4.65 eV), graphene/p-Si (4.2 eV), and MoP NRs/Gr/p-Si (4.8 eV). As the graphene and MoP show metallic properties, the energy band diagrams for the MoP NRs/Gr/p-Si heterostructure are described in Fig. 4d. The large work function difference of 0.45 eV at the graphene/p-Si interface induces a strong band bending, which allows electrons in the conduction band minimum of p-Si to move rapidly through the graphene interlayer**.** For the detailed explanation of band bending at graphene/p-Si contact, band diagrams for the graphene/p-Si contact in three cases (before equilibrium, in equilibrium, in steady-state illumination) are provided in Fig. S9.

### Photoelectrochemical Hydrogen Evolution Reaction

**Fig. 5:**
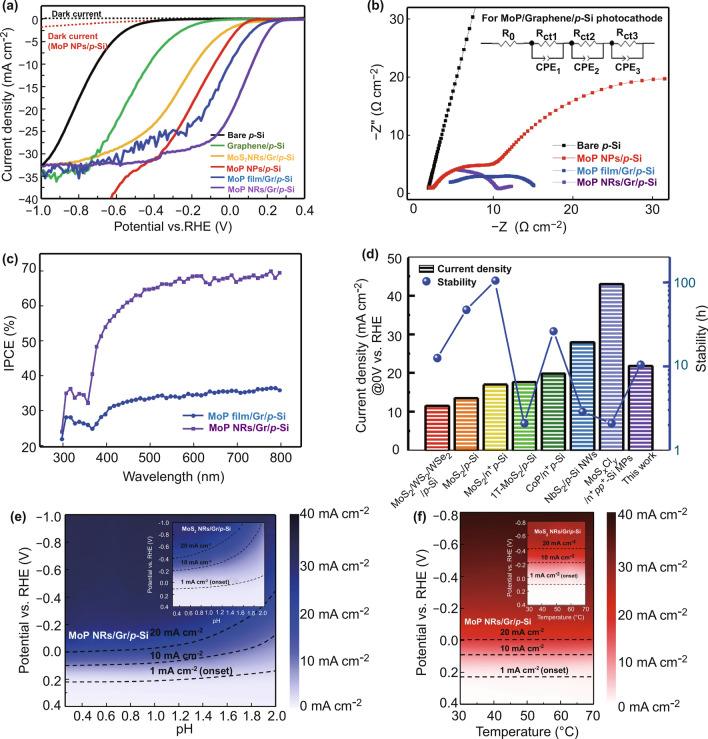
**a** Linear sweep voltammograms (LSVs) of the bare p-Si, graphene/p-Si, MoS_2_ NRs/Gr/p-Si, MoP NPs/p-Si, MoP film/Gr/p-Si, and MoP NRs/Gr/p-Si. **b** Electrochemical impedance spectroscopy (EIS) plots and equivalent circuits (inset). **c** Incident-photon-to-current conversion efficiency (IPCE) measurements of MoP film/Gr/p-Si and MoP NRs/Gr/p-Si. **d** Comparison of current density at 0 V versus RHE and stability between our MoP NRs/Gr/p-Si and previously reported state-of-the-art photocathodes fabricated by the direct method with the high-temperature process. **e** pH- and **f** temperature-dependent current density of MoP NRs/Gr/p-Si and MoS_2_ NRs/Gr/p-Si (inset)

The PEC characterization of MoP NRs/Gr/p-Si photocathode compared to bare p-Si, graphene/p-Si, MoS_2_ NRs/Gr/p-Si, MoP NPs/p-Si, and MoP film/Gr/p-Si is carried out under a simulated air mass 1.5 G condition with a standard three-electrode set-up using 0.5 M H_2_SO_4_ electrolyte. The graphite rod is used as a counter electrode. As shown in current density versus potential (*J-V*) curves in Fig. 5a, the bare p-Si photocathode shows a large negative onset potential because of its catalytic inactivity despite the high electron mobility and suitable bandgap. When MoP nanoparticles are applied to p-Si, catalytic activity is enhanced, resulting in the onset potential shift towards the anodic direction. The onset potential and the photocurrent density at 0 V versus RHE reach the values of 0.08 V versus RHE and 4.8 mA cm^−2^, respectively. However, from potential above − 0.4 V versus RHE, a discernible leakage current is shown due to a low shunt resistance caused by Mo_x_Si layer between the crystalline silicon and MoP nanoparticles [55]. Though no leakage current was observed in MoS_2_/Mo/Mo_x_Si/n^+^p-Si photocathode fabricated by Benck et al. [25], it is clearly due to the low synthesis temperature of 250 ℃. As shown in Fig. S10, the LSV curves of MoP/p-Si obtained at 250 and 900 ℃ evidently show the temperature dependence of leakage current. In Fig. S10a, when synthesized at 250 ℃, a negligible leakage current is observed, whereas it increases dramatically as the temperature rises to 900 ℃ in Fig. S10b.

In this respect, by applying the graphene interlayer, we can completely suppress the formation of the metallic secondary phase as seen that no leakage current is visible with MoP film/Gr/p-Si and MoP NRs/Gr/p-Si photocathodes. In Fig. S11, resistance analysis clearly shows that the leakage current flowing laterally through the silicide layers can be effectively suppressed by applying graphene interlayer. Though the graphene shows no remarkable PEC performance as catalysts, MoP film/Gr/p-Si and MoP NRs/Gr/p-Si photocathodes show the dramatic improvement in PEC performance. Mainly, MoP NRs/Gr/p-Si photocathode exhibits a remarkable catalytic activity for the HER with an onset potential of 0.24 V vs. RHE to reach -1 mA cm^−2^ and a photocurrent density of 21.8 mA cm^−2^ at 0 V versus RHE. The 100 nm thickness of MoP nanorod-arrays is an optimized value to get maximum photocurrent density at 0 V versus RHE. Figure S12 indicates that the photocathode with 100 nm MoP NRs shows the highest photocurrent density at 0 V versus RHE compared to the photocathodes with 70 and 130 nm MoP NRs catalysts.

Electrochemical impedance spectroscopy (EIS) measurements were conducted to understand the PEC performance concerning charge transfer resistance, as shown in Fig. 5b. The obtained EIS data were fitted to a simplified equivalent circuit. The equivalent circuit is composed of charge transfer resistance (*R*_ct_) and constant phase elements (CPEs). The smaller arcs in Nyquist plots indicate the lower charge transfer resistance at each interface. Compared to each semicircle of MoP NPs/p-Si photocathode, the photocathodes to which the graphene interlayer is applied show smaller arcs. Notably, the resistances of MoP NRs/Gr/p-Si photocathode at each interface are remarkably decreased.

The exact values of fitted charge transfer resistance corresponding to each interface are tabulated in Table 1. The *R*_ct,1_ of bare p-Si shows two orders of magnitude higher than the total resistance of other MoP deposited photocathodes. It reflects that molybdenum phosphide enables the electrons to move faster at the interfaces and provides a large number of active sites for energetic kinetics. For the resistances at MoP/electrolyte interface, *R*_ct,3_ of MoP NRs/Gr/p-Si photocathode exhibits the lowest value compared to *R*_ct,3_ of MoP film/Gr/p-Si and *R*_ct,2_ of MoP NPs/p-Si photocathodes. It confirms that for the nanorod-structured MoP, electron transfer to the redox couples is enhanced, which is ascribed to the highly-exposed 1D structure showing the shortest distance towards MoP/electrolyte interface and the largest amount of active sites. Moreover, to identify the synergistic effects of graphene, we compared the resistances at heterointerfaces with and without graphene. The sum of *R*_ct,1_ and *R*_ct,2_ of both the graphene-passivated photocathodes is lower than R_ct,1_ of MoP NPs/p-Si photocathode, which provides compelling evidence that the graphene can act as a highway to help the electrons move from p-Si to MoP catalysts. This result is in good agreement with the formation of Mo-O-C covalent bondings and energetically favorable band bending identified by Raman, FT-IR spectroscopy, and UPS.

**Table 1:** Exact values of fitted charge transfer resistance corresponding to each interface.

Photocathode	Resistance			
	*R*_s_ (Ω cm^2^)	*R*_ct,1_ (Ω cm^2^)	*R*_ct,2_ (Ω cm^2^)	*R*_ct,3_ (Ω cm^2^)
Bare *p*-Si	1.60	3131.13 (*p*-Si → electrolyte)	N/A	N/A
MoP NPs	1.46	16.48 (*p*-Si → MoP NPs)	44.34 (MoP NPs → electrolyte)	N/A
MoP film/Gr	1.47	5.27 (*p*-Si → Graphene)	5.47 (Graphene → MoP film)	4.75 (MoP film → electrolyte)
MoP NRs/Gr	1.12	5.34 *(p*-Si → Graphene)	5.45 (Graphene → MoP NRs)	3.68 (MoP NRs → electrolyte)

The summarized charge transport resistance values of bare p-Si, MoP NPs/p-Si, MoP film/Gr/p-Si, and MoP NRs/Gr/p-Si photocathodes.

The capability of photocathodes to convert the incident photons to electrical current is investigated by the incident photon-to-current conversion efficiency (IPCE) at an applied bias of 0 V versus RHE, as shown in Fig. 5c. The MoP NRs/Gr/p-Si photocathode shows the efficiency of ~ 68% over the entire visible light wavelength, while the MoP film/Gr/p-Si photocathode exhibits the IPCE value of ~ 33% which elucidates that a high quantum efficiency can be attained by nano-structured MoP catalysts.

**Fig. 6:**
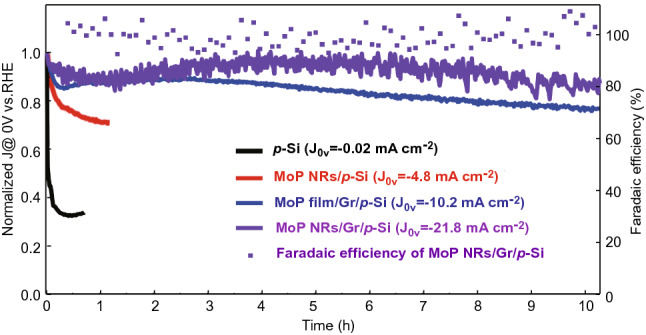
Long-term stability tests for the bare p-Si, MoP NPs/p-Si, MoP film/Gr/p-Si, and MoP NRs/Gr/p-Si photocathodes at 0 V versus RHE and Faradaic efficiency for MoP NRs/Gr/p-Si photocathode

The stability of bare p-Si, MoP NPs/p-Si, MoP film/Gr/p-Si, and MoP NRs/Gr/p-Si photocathodes are evaluated by chronoamperometric measurements in Fig. 6. These measurements are conducted to determine whether MoP catalysts constructed on graphene can act as a passivation layer and maintain their activity without deformation. A critical degradation is shown with a bare p-Si photocathode. Also, in the case of MoP nanoparticles, the photocurrent density of − 4.8 mA cm^−2^ at 0 V versus RHE is not endured 80% of photocurrent density until 1 h. As the catalysts do not fully cover the silicon surface, the formation of an insulating layer is accelerated, hindering charge transfer and finally shutting down the overall activity. On the other hand, it is found that the long-term stability of over 10 h is maintained with the MoP film and MoP nanorod-arrays directly synthesized on graphene. For the MoP NRs/Gr/p-Si photocathode, significantly, the photocurrent degradation is considerably suppressed with retaining over 88% of the performance for 10 h. As shown in Fig. S13, the SEM image reveals that MoP nanorod-arrays show no structural change after a 10-h stability test. Interestingly, it is estimated that the total amount of hydrogen generated at the wafer scale for 10 h is apparently larger than that required for the synthesis of MoP nanorod catalysts, but much lower than that required for the synthesis of graphene in Fig. S13.

The Faradaic efficiency is obtained by conducting a gas chromatography (GC) measurement that collects the evolved hydrogen gas. In Fig. 6, the MoP NRs/Gr/p-Si photocathode shows almost 100% Faradaic yield during the photoelectrochemical process.

To compare the PEC performance of MoP NRs/Gr/p-Si photocathode with that of state-of-the-art photocathodes synthesized by the in-vacuum high-temperature process, photocurrent density at 0 V versus RHE and long-term stability are plotted in Fig. 5d. Most of the photocathodes [22, 24–26, 56] provide a lower photocurrent density at 0 V versus RHE than our photocathode though some of them maintain their catalytic activity for a long time. In contrast, NbS_2_/p-Si NWs [21] and MoS_x_Cl_y_/n^+^pp^+^-Si MPs [23] photocathodes show significantly enhanced photocurrent density while it does not last more than 2 ~ 3 h. However, our photocathode shows a superior PEC performance in balance with photocurrent density and stability. The details about the synthesis method and numerical values are tabulated in Table S1.

For further practical use of MoP NRs/Gr/p-Si photocathode as part of PEC cell, it needs to be operated with high reliability against variations in the pH and temperature of electrolyte. In Fig. 5e, 2D plots show how the current density of MoP NRs/Gr/p-Si and MoS_2_ NRs/Gr/p-Si photocathodes changes at each voltage as the pH changes from 0.26 to 2. The pH of the electrolyte is controlled by mixing 0.5 M H_2_SO_4_ with neutral DI water. In the case of MoS_2_ nanorod-array catalysts (inset), considering the contours with the current density of 1, 10, and 20 mA cm^−2^, the potential required to obtain each current density value dramatically increases as the acidity of electrolyte becomes weaker. On the other hand, the MoP nanorod-arrays catalysts show no definite change up to pH 1.2, and after that, the PEC performance gradually weakened. Similarly, the current density variation depending on electrolyte temperature is plotted in Fig. 5f. As for the temperature effects on the photoelectrodes, it is well known that a net decrease of efficiency is observed with increasing temperature as the reduction in photovoltage induced by radiation losses surpasses an enhancement of interfacial kinetics [57]. However, as shown in Fig. 5f, both samples show high stability against the increase of electrolyte temperature from 30 to 70 °C. It appears that both the incorporated photoelectrochemical catalysts boost the surface reaction enough to alleviate the reduction in the absorber efficiency.

We also investigated how the nanorod structure, which caused a significant increase in PEC performance of MoP having a 3D atomic structure, affects that of MoS_2_ having a 2D atomic structure. MoS_2_ nanorod-arrays and film are synthesized in the same process with MoP nanorod-arrays and MoP film, respectively, except for using sulfur powder. The Raman and XRD spectrums of MoS_2_ nanorods are provided in Fig. S14a, b. As shown in Fig. S14c, there is a noticeable degradation in PEC performance when the nanorod structure is applied to MoS_2_. Besides, it does not maintain its activity for a long time (Fig. S14d). Considering that MoS_2_ has a layered crystal structure held by weak van der Waals force [13], the photogenerated electrons should move through continuous hopping transport at each layer, which causes a large overpotential. Though the nanorod structure contributes to a numerical increment of actives sites, large overpotential hinders an efficient hydrogen evolution reaction.

The electrochemical (EC) HER performances of MoP-based catalysts on graphene/p-Si are also investigated in Fig. S15 and Table S2. To achieve graphene-based direct growth on silicon, highly doped n^++^-Si wafers are used as metallic cathodes. In Fig. S15a, MoP nanorod-array catalysts exhibit a smaller onset potential and overpotential at a current density of 10 mA cm^−2^ compared to MoP film. Also, the Tafel slope for MoP NRs/Gr/n^++^-Si is 97.19 mV per decade, which is lower than the values of 108.99 mV per decade for MoP film/Gr/n^++^-Si, as shown in Fig. S15b. From these result, a larger amount of surface active sites is exposed and faster catalytic activity occurs for MoP nanorods than film electrocatalyst. Since the thickness of our MoP nanorods catalysts is optimized for the PEC system, an enhanced electrocatalytic performance can be obtained by increasing the thickness in the EC system.

**Fig. 7:**
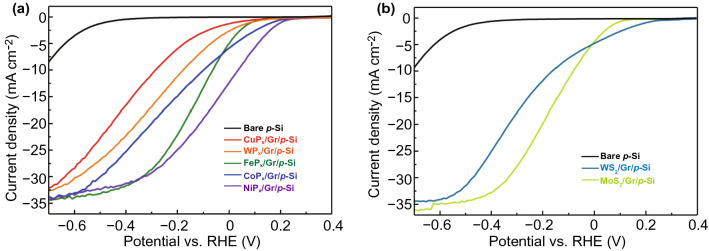
**a** Polarization (*J*-*V*) curves of copper phosphide, tungsten phosphide, iron phosphide, cobalt phosphide, and nickel phosphide deposited photocathodes. **b** Polarization (*J*-*V*) curves of tungsten disulfide and molybdenum disulfide deposited photocathodes

To further demonstrate that the synergistic direct growth on graphene interlayer can be applied to various TMPs and TMDs, we have synthesized CuP_x_, WP_x_, FeP_x_, CoP_x_, NiP_x_, MoS_2_, and WS_2_ on graphene/p-Si substrates. They were prepared by phosphorization of CuO, WO_3_, Fe_2_O_3_, CoO_3_, NiO, and sulfurization of MoO_3_, WO_3_. The synthesis temperature of CuP_x_, CoP_x_, and WS_2_ is 700 ℃, while that of WP_x_, FeP_x_, NiP_x_, and MoS_2_ is 900 ℃. In Fig. 7a, the current density vs. potential (*J-V*) curves of TMPs/Gr/p-Si photocathodes under a simulated air mass 1.5 G condition are shown. For all the TMPs, the onset potentials are shifted toward the anodic direction compared to bare p-Si, indicating that the various TMPs work as efficient PEC catalysts on graphene/p-Si photocathodes. Among them, NiP_x_ and CoP_x_ exhibit a remarkable current density of 12.9 and 6.1 mA cm^−2^ at 0 V vs. RHE, respectively. Also, Fig. 7b presents the current density vs. potential (*J-V*) curves of TMDs/Gr/p-Si photocathodes. Similarly, the onset potentials of MoS_2_/Gr/p-Si and WS_2_/Gr/p-Si photocathodes are shifted toward the anodic direction. If the thickness control or nanostructuring is achieved with these catalysts, the enhanced photoelectrochemical activity will be obtained.

## Conclusions

We successfully demonstrated that MoP nanorod-array catalysts grown on graphene-passivated silicon photocathodes remarkably improve PEC-HER performance in balance with the high photocurrent density of 21.8 mA cm^−2^ at 0 V versus RHE and long-term stability of over 10 h. Through the graphene passivation process, not only the secondary compound which absorbs a parasitic light and provides an inefficient electron path are prevented, but also the synergistic effects of Mo-O-C bondings and strong band bending enhanced the photogenerated electron transport. Benefiting from a large surface area exposing numerous active sites and anti-reflectance of MoP nanorods, the obtained photocathodes exhibited a high quantum efficiency. Furthermore, its high performance was maintained with the increase of pH and temperature. This study provides the framework to rationally design nanostructured PEC catalysts directly on graphene/p-Si photocathodes with high-quality interfaces and superior HER performance.

## Supplementary Information

Supplementary file1 (PDF 1344 kb)
