# Supplementary material for: Direct Synthesis of Molybdenum Phosphide Nanorods on Silicon Using Graphene at the Heterointerface for Efficient Photoelectrochemical Water Reduction
Source: Nanomicro Lett. 2021 Mar 1;13:81. doi: 10.1007/s40820-021-00605-7 (PMC8006559; doi:10.1007/s40820-021-00605-7)
Supplement: Supplementary file 1 — Supplementary file1 (PDF 1344 kb) [file 40820_2021_605_MOESM1_ESM.pdf]

Supporting Information for

Direct Synthesis of Molybdenum Phosphide Nanorods on Silicon Using Graphene at the Heterointerface for Efficient Photoelectrochemical Water Reduction

Sang Eon Jun[1], Seokhoon Choi[1], Shinyoung Choi[2], Tae Hyung Lee[1], Changyeon Kim[1], Jin Wook Yang[1], Woon-Oh Choe[1], In-Hyuk Im[1], Cheol-Joo Kim[2,]*, Ho Won Jang[1,]*

[1]Department of Materials Science and Engineering, Research Institute of Advanced Materials, Seoul National University, Seoul 08826, Republic of Korea

[2]Department of Chemical Engineering, Pohang University of Science and Technology, Pohang 37673, Republic of Korea

*Corresponding authors. E-mail: hwjang@snu.ac.kr (H. W. Jang), kimcj@postech.ac.kr (C.-J. Kim)

Supplementary Figures and Tables

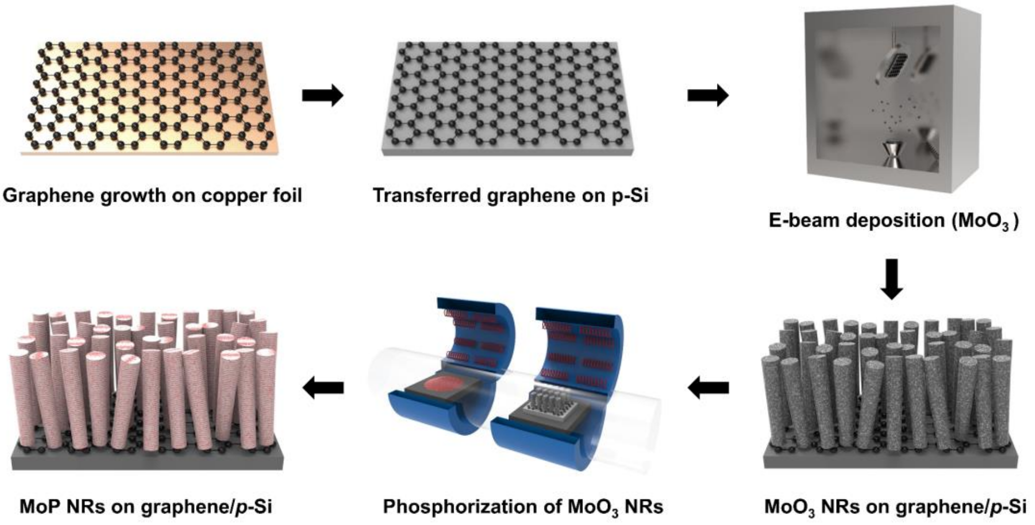

Fig. S1 Detailed schematic illustration for the synthesis of MoP NRs/Gr/p-Si photocathode

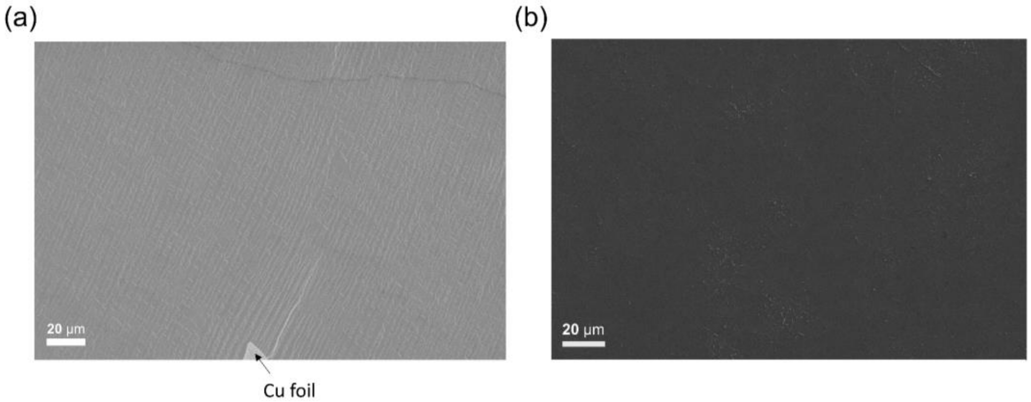

Fig. S2 SEM images of graphene (**a**) grown on copper foil and (**b**) transferred to p-Si

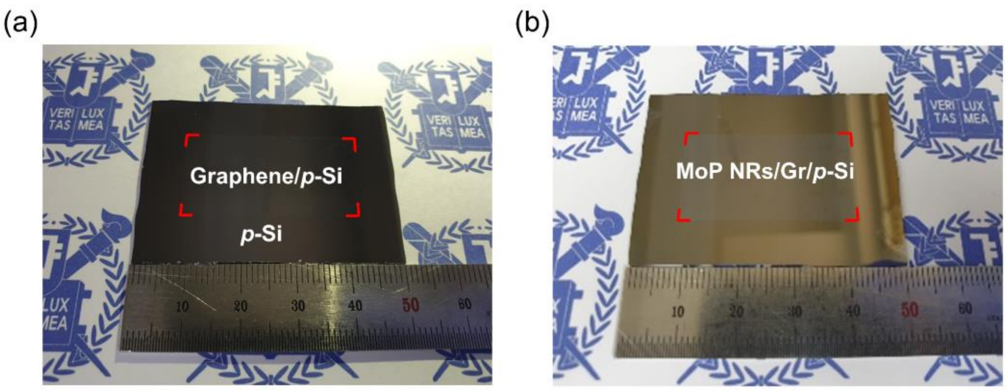

Fig. S3 Photographic images of (a) graphene/p-Si wafer and (b) MoP NRs/graphene/p-Si wafer

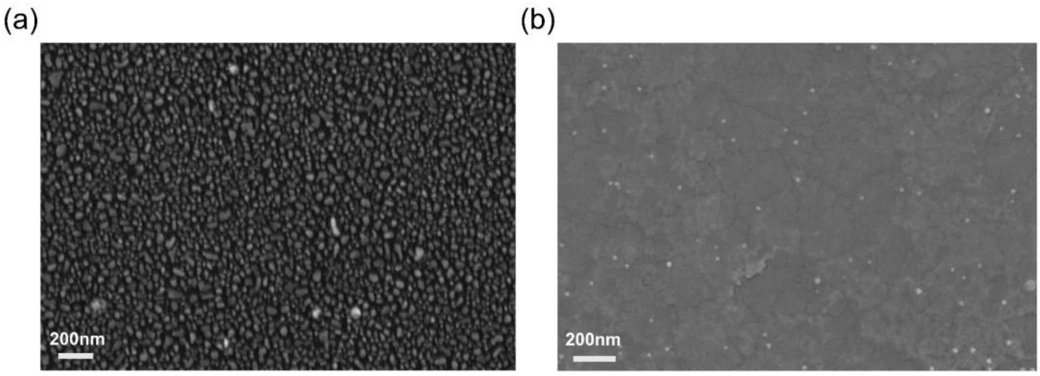

Fig. S4 SEM images of (**a**) MoP nanoparticles grown on p-Si and (**b**) MoP film grown on graphene/p-Si

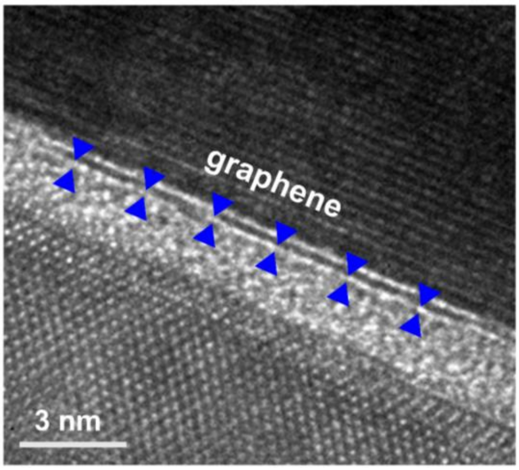

Fig. S5 Zoom-in cross-sectional HR-TEM image of MoP NRs/graphene/SiOx/p-Si showing a graphene interlayer

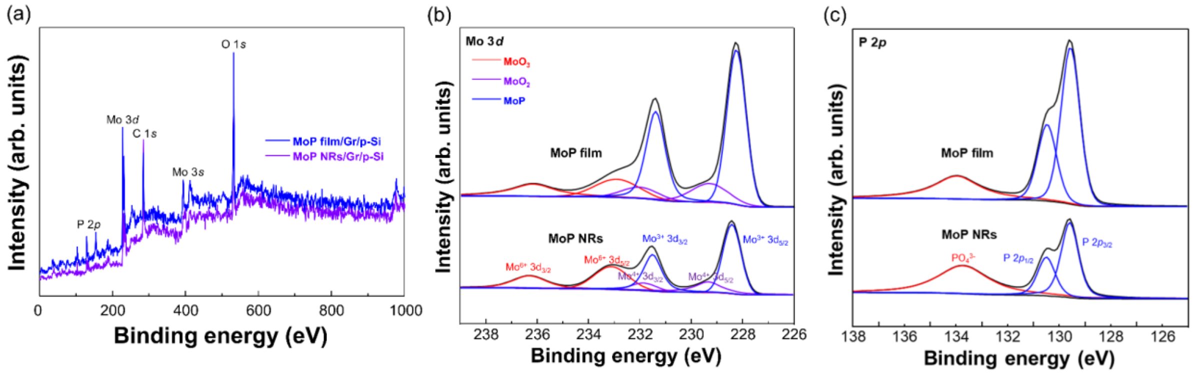

Fig. S6 (**a**) XPS wide scans of MoP film/Gr/p-Si and MoP NRs/Gr/p-Si. XPS core-level spectra of (**b**) Mo 3*d*, and (**c**) P 2*p*

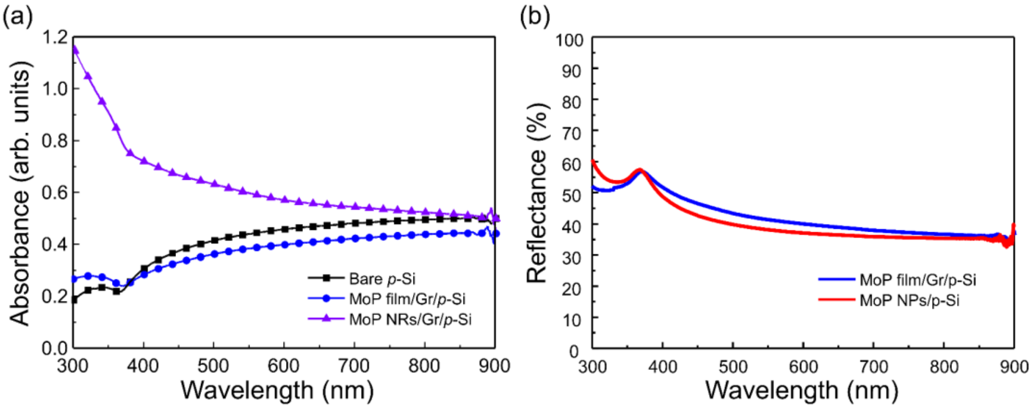

Fig. S7 (**a**) Absorbance vs. wavelength spectra of bare p-Si, MoP film/Gr/p-Si, and MoP NRs/Gr/p-Si (**b**) Reflectance vs. wavelength spectra of MoP film/Gr/p-Si and MoP NPs/p-Si

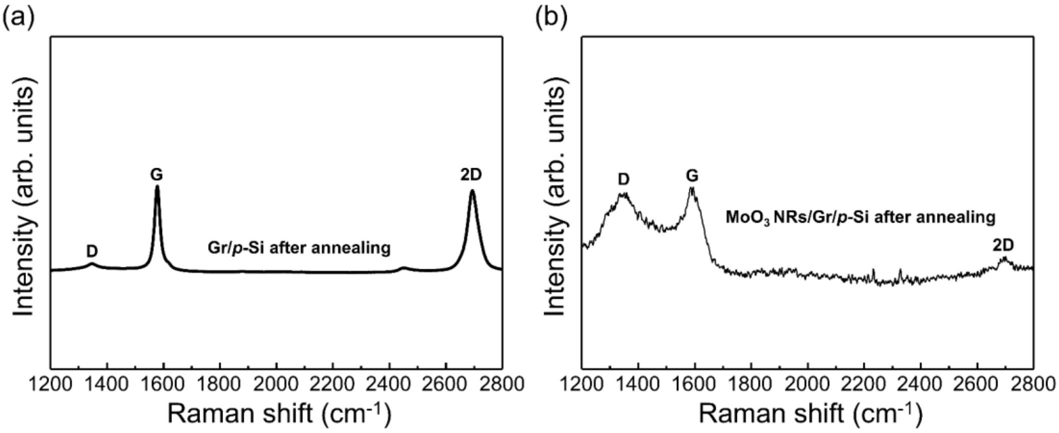

Fig. S8 Raman spectra of (**a**) Gr/p-Si and (**b**) MoO_3 NRs/Gr/p-Si after annealing at 900 °C in H_2/N_2 atmosphere

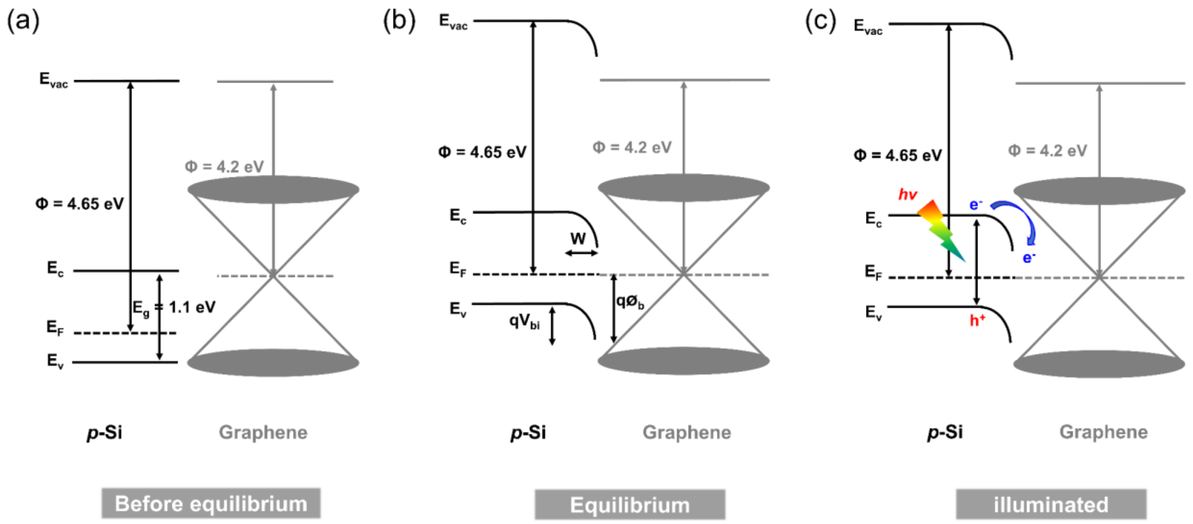

Fig. S9 The energy band diagram of p-Si/graphene contact in three cases. (**a**) before equilibrium (**b**) in equilibrium (**c**) in steady-state illumination

Before equilibrium, p-type silicon and graphene have the work function of 4.65 eV and 4.2 eV, respectively, which have been derived from UPS. By forming a Schottky junction between p-Si and graphene, electrons will flow until equilibrium, which leads to the occurrence of an interfacial electric field [S1, S2]. qV_{bi} is the built-in potential, $q\o_b$ is the Schottky barrier, and W is the depletion width. As a result, energetically favorable band bending is formed at the interface. Under steady-state illumination, electron-hole pairs are generated and the movement of photogenerated electrons is accelerated by band bending. Moreover, the charge carrier lifetime increases as the band bending enhances the charge separation and retards carrier recombination.

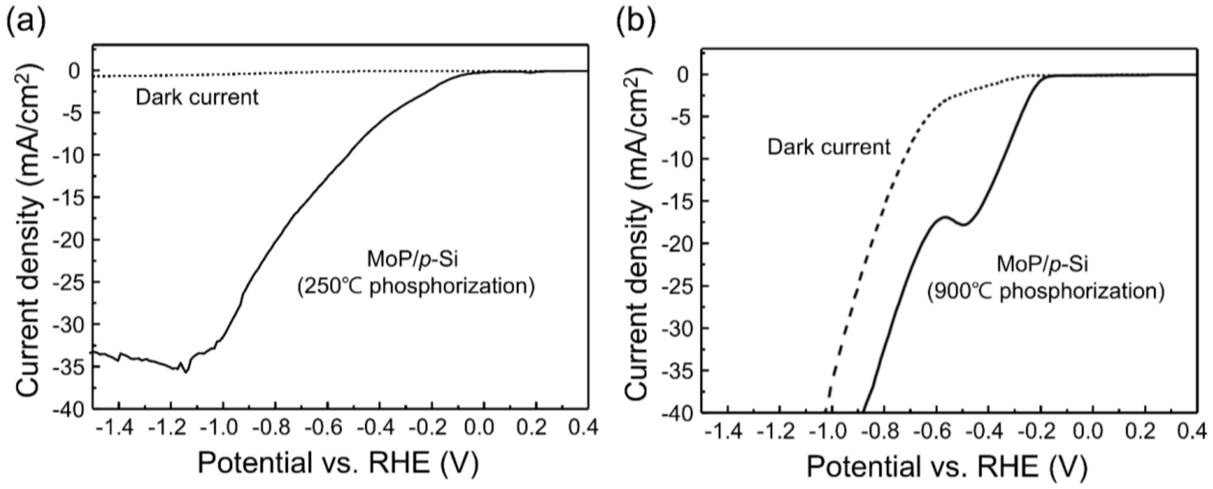

Fig. S10 LSV curves of MoP/p-Si photocathodes at (**a**) 250 ℃ phosphorization and (**b**) 900 ℃ phosphorization for the leakage current dependence on the synthesis temperature

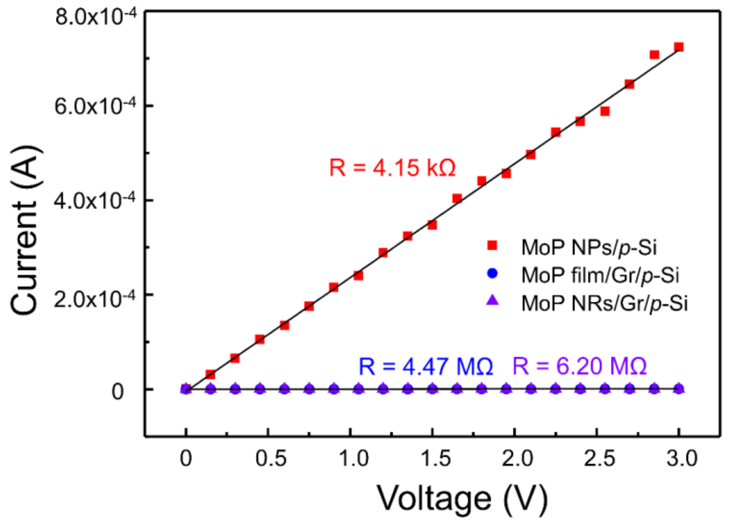

Fig. S11 I-V plots for surface resistance of MoP NPs/p-Si, MoP film/Gr/p-Si and MoP NRs/Gr/p-Si

The resistance analysis is conducted by measuring the current flowing on the surface of silicon photocathodes depending on the applied voltage to prove that the graphene interlayer can suppress the leakage current. As for MoP NPs/p-Si, the surface of device shows a certain amount of current with the resistance of 4.15 kΩ. On the other hand, negligible electric current was exhibited by MoP film/Gr/p-Si and MoP NRs/Gr/p-Si with 4.47 MΩ and 6.20 MΩ, respectively. From this result, it is confirmed that the graphene between p-Si and MoP can effectively prevent the formation of conducting silicide layer and suppress the leakage current.

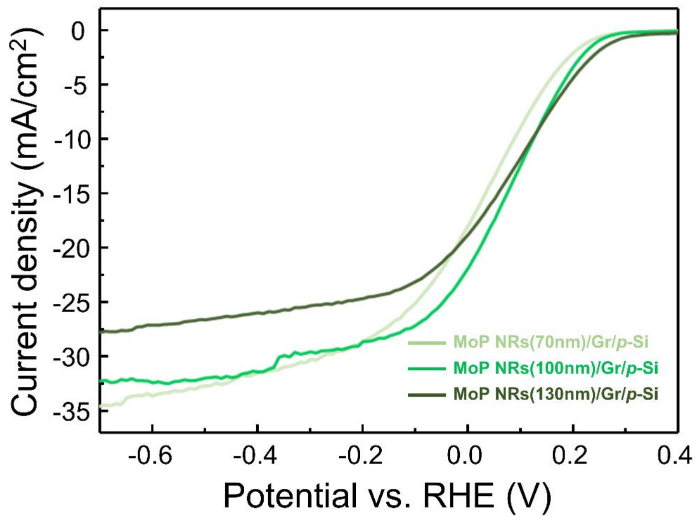

Fig. S12 LSVs of 70nm, 100nm, and 130nm-thickness MoP nanorods synthesized on graphene-passivated p-type silicon photocathodes

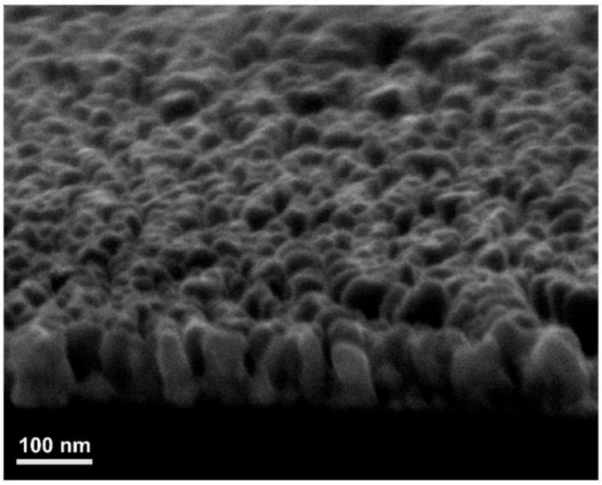

Fig. S13 SEM image of MoP NRs/Gr/p-Si after 10 hours long-term stability test and total amount of hydrogen generated in 10 hours compared to the total amount of hydrogen required for the synthesis

<Total amount of hydrogen generated in 10 hours>

* Total amount of charges obtained by integrating the time on the x-axis and current density on the y-axis in I-t graph (Fig. 6)

= Q = 724.334 C/cm^2

* Total number of moles per square centimeter

= n = Q/Fz = 724.334 C/cm^2 / (96485 C/mol * 2) = 0.00375361 mol/cm^2

* Total volume per square centimeter = V = nRT/P = (0.003754 mol/cm^2 * 0.082 L·atm/mol * 298 K) / 1 atm = 91.733 mL/cm^2

* Total volume per 4-inch (100 mm) silicon wafer = 91.733 mL/cm^2 * (5 * 5 * 3.14 cm^2) = 7201 mL

<Total amount of hydrogen required for the synthesis>

- Graphene synthesis

* Hydrogen flux = 70 sccm = 70 mL/min

* Time of synthesis = 8 hours = 480 min

* Total volume of hydrogen = 70 mL/min * 480 min = 33600 mL

- MoP nanorods synthesis

* Hydrogen flux = 100 sccm = 100 mL/min

* Time of synthesis = 30 min

* Total volume of hydrogen = 100 mL/min * 30 min = 3000 mL

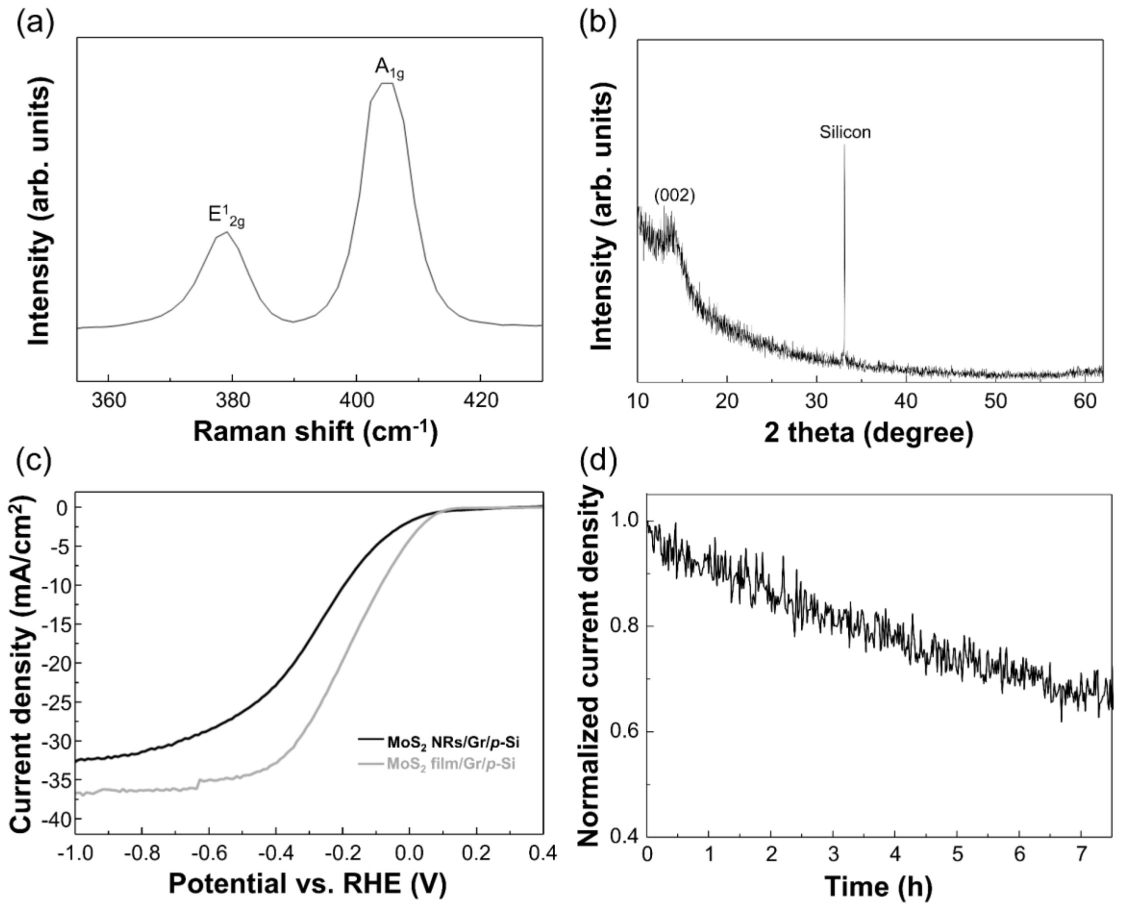

Fig. S14 Characterization and PEC performance of MoS_2 nanorods(100nm)/Gr/p-Si photocathode. (**a**) Raman spectrum of MoS_2 nanorods(100nm) (**b**) XRD spectrum (**c**) LSVs of MoS_2 nanorods(100nm)/Gr/p-Si compared to that of MoS_2 film/Gr/p-Si photocathode. (**d**) Long-term stability tests at 0V vs. RHE

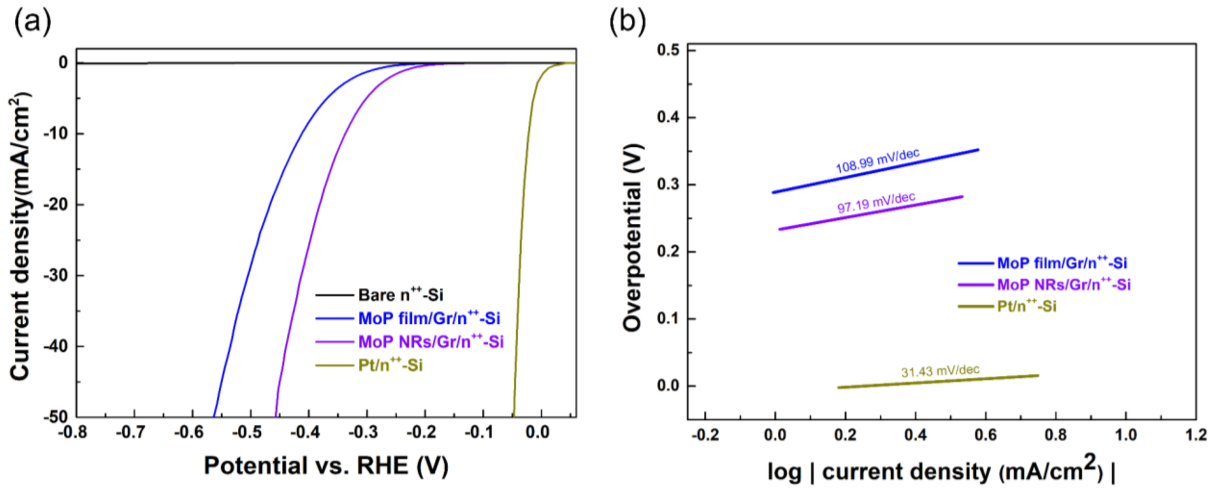

Fig. S15 Electrochemical (EC) characterizations of the fabricated photocathodes with IR-correction. (**a**) LSVs of the bare n^{++}-Si, MoP film/Gr/n^{++}-Si, MoP NRs/Gr/n^{++}-Si, and Pt film/n^{++}-Si cathodes (**b**) Tafel plots of each sample plotted as logarithmic (*j*) vs. potential RHE

Table S1 Comparison of the characterizations between our MoP NRs/Gr/p-Si and previously reported state-of-the-art photocathodes fabricated by the direct method with the vacuum process

No.	Catalysts	Synthesis method	Photocathode	Current density @ 0V vs. RHE	Stability (@ 0 V vs. RHE)	Refs.
1	$MoS_2/WS_2/WSe_2$ film	PLD (MoS_2, WS_2, WSe_2)	*p*-Si	11.5 mA/cm^2	12 h @ -0.2 V	[S3]
2	MoS_2 film	Thermolysis (MoO_3 + $(NH_4)_2MoS_2$, S)	*p*-Si	13.5 mA/cm^2	45 h	[S4]
3	MoS_2 film	Sulfurization (Mo, S)	n^+p-Si	17 mA/cm^2	100 h	[S5]
4	1T-MoS_2 film	CVD ($MoCl_5$, S)	*p*-Si	17.6 mA/cm^2	2 h	[S6]
5	CoP film	Phosphorization (Co, P)	n^+p-Si	19.8 mA/cm^2	25 h	[S7]
6	NbS_2	CVD ($NbCl_5$, S\)	*p*-Si NWs	28 mA/cm^2	10000 s	[S8]
7	MoS_xCl_y film	CVD ($MoCl_5$, S)	n^+pp^+-Si MPs	43 mA/cm^2	2 h	[S9]
Our work	MoP NRs/Gr	Phosphorization (MoO_3, P)	*p*-Si	19.5 mA/cm^2	10 h	-

Table S2 Electrochemical performance of MoP film/Gr/n^{++}-Si, MoP NRs/Gr/n^{++}-Si, and Pt/n^{++}-Si cathodes

Electrodes	Potential (V) @ 10 mA/cm^2	Potential (V) @ 50 mA/cm^2	Tafel slope (mV/dec)
MoP film/Gr	-0.41	-0.56	108.99
MoP NRs/Gr	-0.33	-0.45	97.19
Pt	-0.02	-0.03	31.43

Supplementary References

[S1]Y. An, A. Behnam, E, Pop, A. Ural, Metal-semiconductor-metal photodetectors based on graphene/p-type silicon Schottky junctions. Appl. Phys. Lett. **102**, 013110 (2013). https://doi.org/10.1063/1.4773992

[S2]M. Casalino, Silicon meets graphene for a new family of near-infrared schottky photodetectors. Appl. Sci. **9**, 3677 (2019). https://doi.org/10.3390/app9183677

[S3]S. Seo, S. Kim, H. Choi, J. Lee, H. Yoon et al., Direct in situ growth of centimeter-scale multi-heterojunction $MoS_2/WS_2/WSe_2$ thin-film catalyst for photo-electrochemical

hydrogen evolution. Adv. Sci. **6**, 1900301 (2019). https://doi.org/10.1002/advs.201900301

[S4]A. Hasani, Q. Van Le, M. Tekalgne, M.-J. Choi, T. H. Lee et al., Direct synthesis of two-dimensional MoS_2 on p-type Si and application to solar hydrogen production. NPG Asia Mater. **11**, 1-9 (2019). https://doi.org/10.1038/s41427-019-0145-7

[S5]J. D. Benck, S. C. Lee, K. D. Fong, J. Kibsgaard, R. Sinclair et al., Designing active and stable silicon photocathodes for solar hydrogen production using molybdenum sulfide nanomaterials. Adv. Energy Mater. **4**, 1400739 (2014). https://doi.org/10.1002/aenm.201400739

[S6]Q. Ding, F. Meng, C. R. English, M. Cabán-Acevedo, M. J. Shearer et al., Efficient photoelectrochemical hydrogen generation using heterostructures of Si and chemically exfoliated metallic MoS_2. J. Am. Chem. Soc. **136**, 8504-8507 (2014). https://doi.org/10.1021/ja5025673

[S7]T. R. Hellstern, J. D. Benck, J. Kibsgaard, C. Hahn, T. F. Jaramillo, Engineering cobalt phosphide (CoP) thin film catalysts for enhanced hydrogen evolution activity on silicon photocathodes. Adv. Energy Mater. **6**, 1501758 (2016). https://doi.org/10.1002/aenm.201501758

[S8]P. Gnanasekar, D. Periyanagounder, P. Varadhan, J.-H. He, J. Kulandaivel, Highly efficient and stable photoelectrochemical hydrogen evolution with 2D-NbS_2/Si nanowire heterojunction. ACS Appl. Mater. Interfaces **11**, 44179-44185 (2019). https://doi.org/10.1021/acsami.9b14713

[S9]Q. Ding, J. Zhai, M. Cabán-Acevedo, M. J. Shearer, L. Li et al., Designing Efficient Solar-Driven Hydrogen Evolution Photocathodes Using Semitransparent MoQ_xCl_y (Q= S, Se) Catalysts on Si Micropyramids. Adv. Mater. **27**, 6511-6518 (2015). https://doi.org/10.1002/adma.201501884
